# Crystal structure of ethyl 6-(2-fluoro­phen­yl)-4-hy­droxy-2-sulfanyl­idene-4-tri­fluoro­meth­yl-1,3-diazinane-5-carboxyl­ate

**DOI:** 10.1107/S2056989015005836

**Published:** 2015-04-02

**Authors:** M. S. Krishnamurthy, Noor Shahina Begum

**Affiliations:** a Department of Studies in Chemistry, Central College Campus, Bangalore University, Bangalore 560 001, Karnataka, India

**Keywords:** crystal structure, di­hydro­pyrimidine derivative, organofluorine compounds, hydrogen bonding

## Abstract

In the title compound, C_14_H_14_F_4_N_2_O_3_S, the central di­hydro­pyrimidine ring adopts a sofa conformation with the C atom bearing the 2-fluoro­benzene ring displaced by 0.596 (3) Å from the other five atoms. The 2-fluoro­benzene ring is positioned axially and bis­ects the pyrimidine ring with a dihedral angle of 70.92 (8)°. The mol­ecular conformation is stabilized by an intra­molecular O—H⋯O hydrogen bond, generating an *S*(6) ring. The crystal structure features C—H⋯F, N—H⋯S and N—H⋯O hydrogen bonds, which link the mol­ecules into a three-dimensional network.

## Related literature   

For the bioactivity of di­hydro­pyrimidines, see: Atwal *et al.* (1989 ▸); Brier *et al.* (2004 ▸); Cochran *et al.* (2005 ▸); Moran *et al.* (2007 ▸); Zorkun *et al.* (2006 ▸). For the bioactivity of organofluorine compounds, see: Hermann *et al.* (2003 ▸); Ulrich (2004 ▸). For related structures, see: Mosslemin *et al.* (2009 ▸); Li *et al.* (2011 ▸); Huang *et al.* (2012 ▸).
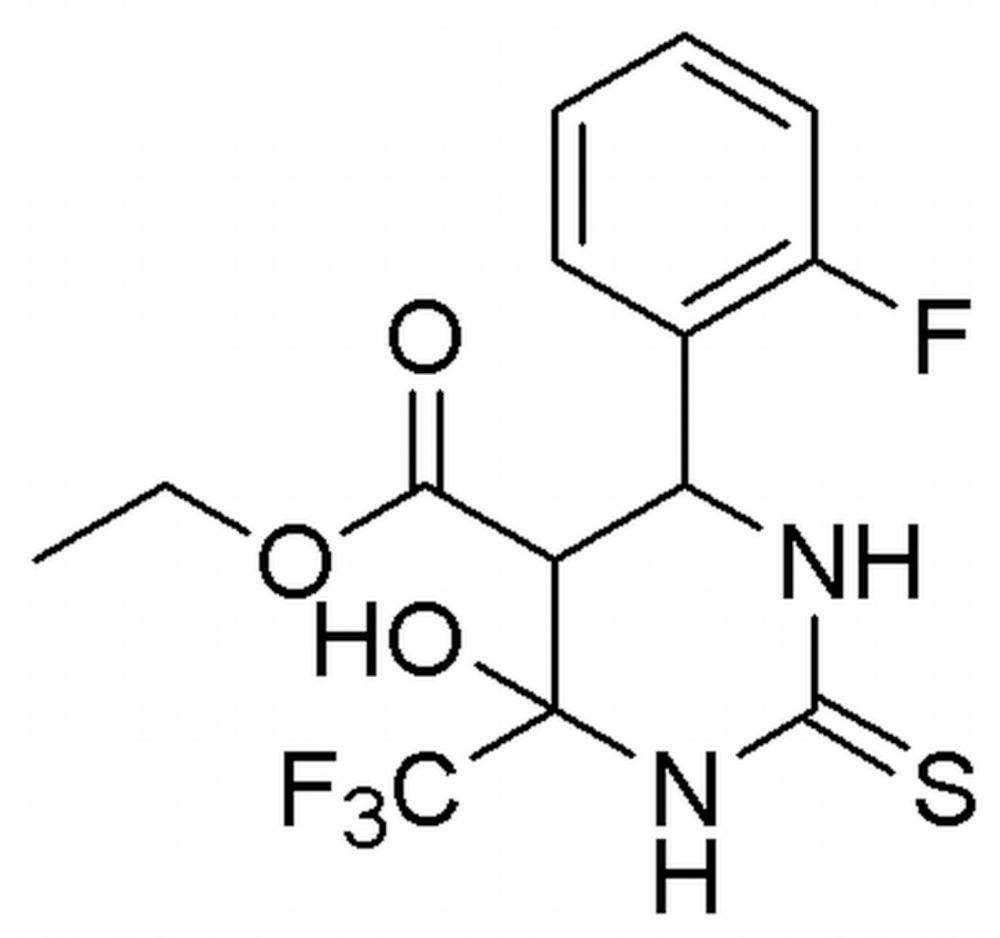


## Experimental   

### Crystal data  

C_14_H_14_F_4_N_2_O_3_S
*M* *_r_* = 366.33
Monoclinic, 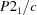

*a* = 10.937 (3) Å
*b* = 9.934 (3) Å
*c* = 14.629 (4) Å
β = 108.239 (5)°
*V* = 1509.7 (8) Å^3^
*Z* = 4
Mo *K*α radiation
μ = 0.28 mm^−1^
*T* = 100 K
0.15 × 0.12 × 0.09 mm

### Data collection  

Bruker SMART APEX CCD detector diffractometer
Absorption correction: multi-scan (*SADABS*; Bruker, 1998 ▸) *T*_min_ = 0.952, *T*_max_ = 0.957
7619 measured reflections
2646 independent reflections
2138 reflections with *I* > 2σ(*I*)
*R*_int_ = 0.034

### Refinement  

*R*[*F* ^2^ > 2σ(*F* ^2^)] = 0.046
*wR*(*F* ^2^) = 0.141
*S* = 1.03
2646 reflections
219 parameters
H-atom parameters constrained
Δρ_max_ = 0.64 e Å^−3^
Δρ_min_ = −0.29 e Å^−3^

### 

Data collection: *SMART* (Bruker,1998 ▸); cell refinement: *SAINT-Plus* (Bruker, 1998 ▸); data reduction: *SAINT-Plus*; program(s) used to solve structure: *SHELXS97* (Sheldrick, 2008 ▸); program(s) used to refine structure: *SHELXL97* (Sheldrick, 2008 ▸); molecular graphics: *ORTEP-3 for Windows* (Farrugia, 2012 ▸) and *CAMERON* (Watkin *et al.*, 1996 ▸); software used to prepare material for publication: *WinGX* (Farrugia, 2012 ▸).

## Supplementary Material

Crystal structure: contains datablock(s) global, I. DOI: 10.1107/S2056989015005836/hb7389sup1.cif

Structure factors: contains datablock(s) I. DOI: 10.1107/S2056989015005836/hb7389Isup2.hkl

Supporting information file. DOI: 10.1107/S2056989015005836/hb7389Isup3.cml

**Fig. 1.**
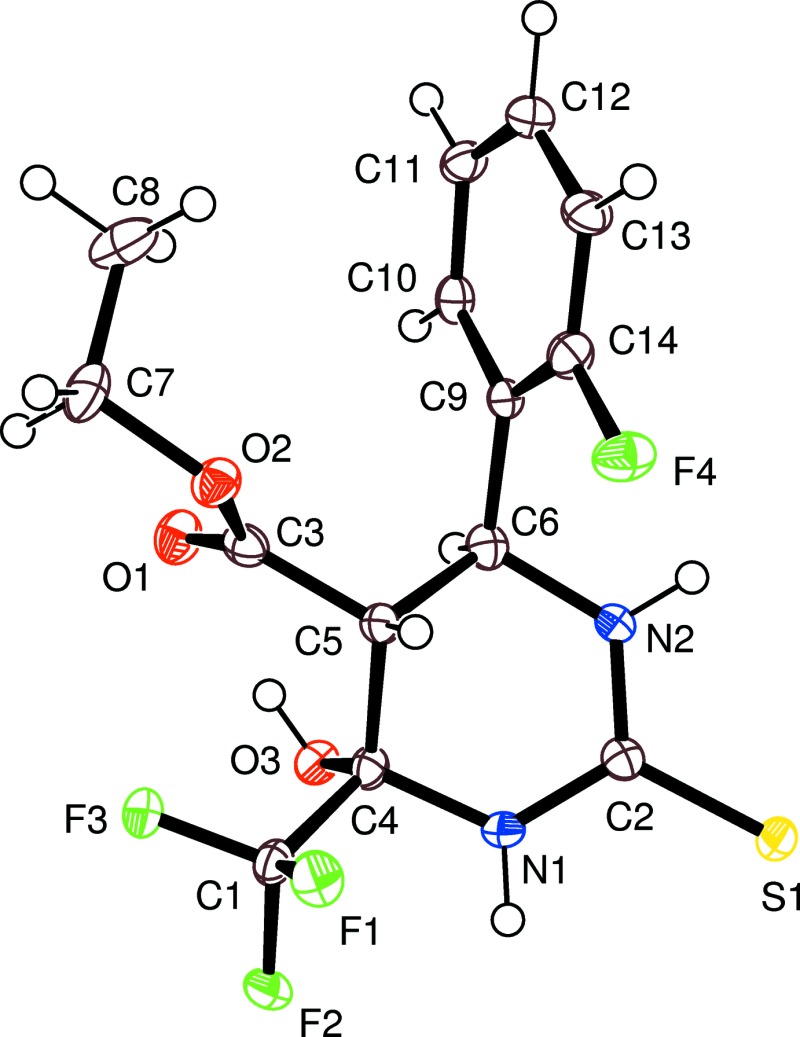
The molecular structure of the title compound with displacement ellipsoids drawn at the 50% probability level.

. DOI: 10.1107/S2056989015005836/hb7389fig1.tif
The mol­ecular structure of the title compound with displacement ellipsoids drawn at the 50% probability level.

**Fig. 2.**
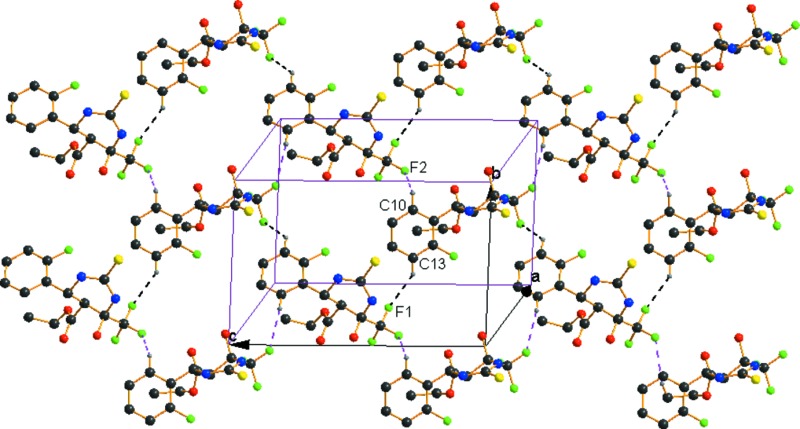
Unit-cell packing of the title compound showing C—H···F interactions as dotted lines. H atoms not involved in hydrogen bonding have been excluded.

. DOI: 10.1107/S2056989015005836/hb7389fig2.tif
Unit-cell packing of the title compound showing C—H⋯F inter­actions as dotted lines. H atoms not involved in hydrogen bonding have been excluded.

**Fig. 3.**
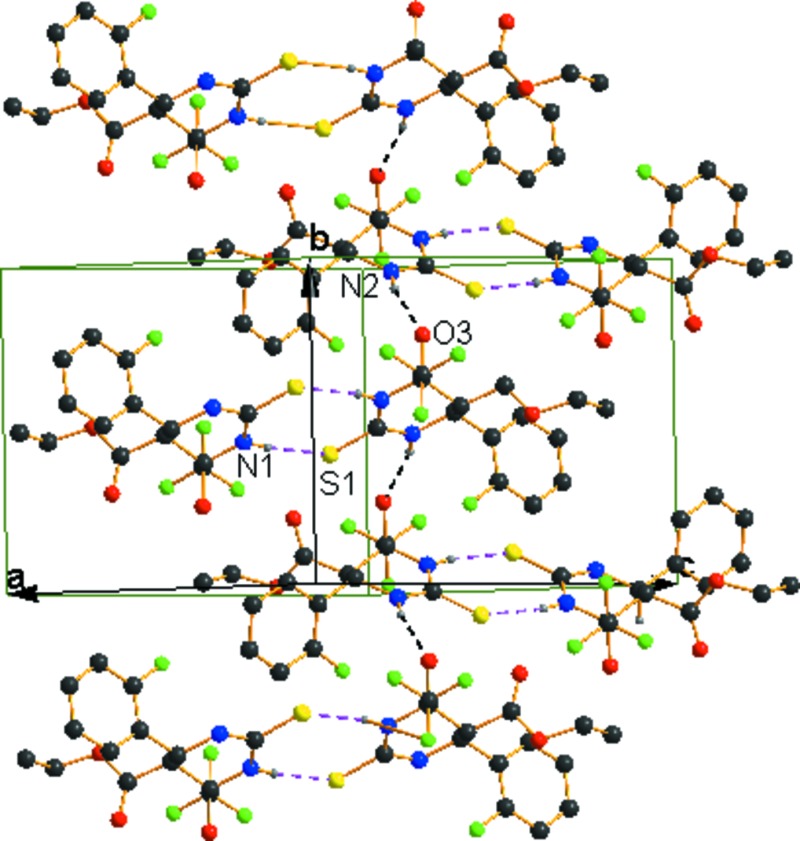
Unit-cell packing depicting the N—H···S and N—H···O interactions with dotted lines. H atoms not involved in hydrogen bonding have been excluded.

. DOI: 10.1107/S2056989015005836/hb7389fig3.tif
Unit-cell packing depicting the N—H⋯S and N—H⋯O inter­actions with dotted lines. H atoms not involved in hydrogen bonding have been excluded.

CCDC reference: 1055499

Additional supporting information:  crystallographic information; 3D view; checkCIF report

## Figures and Tables

**Table 1:** Hydrogen-bond geometry (, )

*D*H*A*	*D*H	H*A*	*D* *A*	*D*H*A*
O3H3O1	0.84	2.07	2.787(3)	143
C10H10F2^i^	0.95	2.62	3.285(6)	128
C13H13F1^ii^	0.95	2.56	3.262(8)	131
N1H1S1^iii^	0.88	2.52	3.389(2)	171
N2H2O3^iv^	0.88	2.23	3.079(3)	162

Symmetry codes: (i) 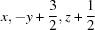; (ii) 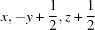; (iii) 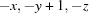; (iv) 
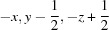
.

## supplementary crystallographic information

### S1. Comment

Dihydropyrimidine (DHPM) derivatives can be used as potential calcium channel blockers (Zorkun *et al.*, 2006), inhibitors of mitotic kinesin Eg5 for treating cancer (Cochran *et al.*, 2005) and as TRPA1 modulators for treating pain (Moran *et al.*, 2007). In addition, compounds that contain fluorine have special bioactivity, *e.g.* flumioxazin is a widely used herbicide (Hermann *et al.*, 2003; Ulrich, 2004). One of the most popular fluorine- containing functional groups in drug molecules is the trifluoromethyl moiety. Because it contains three fluorine atoms, it exerts significant changes on neighbouring groups, such as increasing the acidity of other centers nearby. It is also one of the most lipophilic groups known, so it provides an extremely useful way of making a molecule more easily delivered to the active site in the body. This led us to focus our attention on the synthesis and bioactivity of these important fused perfluoroalkylated heterocyclic compounds. During the synthesis of DHPM derivatives, the title compound, an intermediate C_14_H_14_F_4_N_2_O_3_S (I) was isolated and the structure confirmed by X-ray diffraction. The molecular structure of the title compound is shown in Fig. 1. The 2-fluoro phenyl ring at chiral carbon atom C6 is positioned axially and bisects the pyrimidine ring with a dihedral angle of 70.92 (8) °. The hexahydro pyrimidine ring adopts a sofa conformation with atom C6 displaced by -0.5961 (3) Å from the mean plane of the other five atoms (N1/C2/N2/C5/C4). The carbonyl group of the exocyclic ester at C5 adopts a *cis* orientation with respect to C5—C4 single bond. The 2-fluoro phenyl ring adopts an *anti* periplanar conformation with respect to C6—H6 bond of the pyrimidine ring. The molecular structure is stabilized by intramolecular O—H···O hydrogen bond, generating an S(6) ring. The crystal structure is primarily stabilized by intermolecular C10—H10···F2 and C13—H13···F1 interactions which result in two dimensional sheets along [011] (Table. 1; Fig. 2). The packing is further stabilized by intermolecular N—H···S hydrogen bonds resulting in a centrosymmetric head to head dimer with graph set *R*^2^_2_(8) notation (Bernstein *et al.*, 1995) and intermolecular N—H···O hydrogen bonds form a molecular chain along the crystallographic *b* axis (Table. 1; Fig. 3).

### S2. Experimental

The title compound was synthesized by the reaction of 2-fluorobenzaldehyde (1.24 g, 10 mmol), ethyl 4, 4, 4, trifluoroacetoacetate (2.21 g, 12 mmol) and thiourea (1.14 g, 15 mmol) in 15 ml ethanol was refluxed for 6 h in the presence of concentrated hydrochloric acid as a catalyst. The reaction was monitored with TLC and the reaction medium was quenched in ice cold water. The precipitate obtained was filtered and dried. The compound was recrystallized from ethanol solvent by slow evaporation method, yielding colorless blocks (yield 82%; m.p. 455 K).

### S3. Refinement

Hydrogen atoms involved in hydrogen-bonding interactions were located by difference methods and their positional and isotropic displacement parameters were refined. Other H atoms were placed in calculated positions, with C—H (aromatic) = 0.95 ° A and C—H (aliphatic) = 0.98 ° A, 0.99 ° A or 1.00 ° A, and treated as riding, with *U*_iso_(H) = 1.2*U*_eq_(C).

### Crystal data

**Table:**

C_14_H_14_F_4_N_2_O_3_S	*F*(000) = 752
*M**_r_* = 366.33	*D*_x_ = 1.612 Mg m^−^^3^
Monoclinic, *P*2_1_/*c*	Mo *K*α radiation, λ = 0.71073 Å
Hall symbol: -P 2ybc	Cell parameters from 2646 reflections
*a* = 10.937 (3) Å	θ = 2.0–25.0°
*b* = 9.934 (3) Å	µ = 0.28 mm^−^^1^
*c* = 14.629 (4) Å	*T* = 100 K
β = 108.239 (5)°	Block, colourless
*V* = 1509.7 (8) Å^3^	0.15 × 0.12 × 0.09 mm
*Z* = 4	

### Data collection

**Table:**

Bruker SMART APEX CCD detector diffractometer	2646 independent reflections
Radiation source: fine-focus sealed tube	2138 reflections with *I* > 2σ(*I*)
Graphite monochromator	*R*_int_ = 0.034
ω scans	θ_max_ = 25.0°, θ_min_ = 2.0°
Absorption correction: multi-scan (*SADABS*; Bruker, 1998)	*h* = −12→13
*T*_min_ = 0.952, *T*_max_ = 0.957	*k* = −11→7
7619 measured reflections	*l* = −17→17

### Refinement

**Table:**

Refinement on *F*^2^	Primary atom site location: structure-invariant direct methods
Least-squares matrix: full	Secondary atom site location: difference Fourier map
*R*[*F*^2^ > 2σ(*F*^2^)] = 0.046	Hydrogen site location: inferred from neighbouring sites
*wR*(*F*^2^) = 0.141	H-atom parameters constrained
*S* = 1.03	*w* = 1/[σ^2^(*F*_o_^2^) + (0.0939*P*)^2^ + 0.0341*P*] where *P* = (*F*_o_^2^ + 2*F*_c_^2^)/3
2646 reflections	(Δ/σ)_max_ < 0.001
219 parameters	Δρ_max_ = 0.64 e Å^−^^3^
0 restraints	Δρ_min_ = −0.29 e Å^−^^3^

### Special details

**Table:**

Geometry. All s.u.'s (except the s.u. in the dihedral angle between two l.s. planes) are estimated using the full covariance matrix. The cell s.u.'s are taken into account individually in the estimation of s.u.'s in distances, angles and torsion angles; correlations between s.u.'s in cell parameters are only used when they are defined by crystal symmetry. An approximate (isotropic) treatment of cell s.u.'s is used for estimating s.u.'s involving l.s. planes.
Refinement. Refinement of *F*^2^ against ALL reflections. The weighted *R*-factor *wR* and goodness of fit *S* are based on *F*^2^, conventional *R*-factors *R* are based on *F*, with *F* set to zero for negative *F*^2^. The threshold expression of *F*^2^ > 2σ(*F*^2^) is used only for calculating *R*-factors(gt) *etc*. and is not relevant to the choice of reflections for refinement. *R*-factors based on *F*^2^ are statistically about twice as large as those based on *F*, and *R*- factors based on ALL data will be even larger.

### Fractional atomic coordinates and isotropic or equivalent isotropic displacement parameters (Å^2^)

**Table:**

	*x*	*y*	*z*	*U*_iso_*/*U*_eq_	
S1	0.09623 (6)	0.40020 (7)	0.12780 (4)	0.0190 (2)	
O1	−0.35850 (17)	0.71225 (18)	0.25205 (13)	0.0243 (5)	
O2	−0.44764 (17)	0.50537 (18)	0.22450 (13)	0.0214 (4)	
O3	−0.18273 (16)	0.75721 (18)	0.15250 (12)	0.0194 (4)	
H3	−0.2203	0.7777	0.1926	0.029*	
N1	−0.10705 (19)	0.5616 (2)	0.09977 (14)	0.0177 (5)	
H1	−0.0968	0.5790	0.0436	0.021*	
N2	−0.0313 (2)	0.4589 (2)	0.24785 (14)	0.0178 (5)	
H2	0.0244	0.4048	0.2875	0.021*	
F1	−0.36294 (14)	0.50800 (15)	−0.00967 (10)	0.0242 (4)	
F2	−0.29456 (14)	0.70124 (16)	−0.04041 (10)	0.0238 (4)	
F3	−0.42992 (13)	0.68945 (15)	0.03915 (10)	0.0225 (4)	
F4	−0.18118 (15)	0.23990 (15)	0.27621 (10)	0.0270 (4)	
C1	−0.3263 (2)	0.6310 (3)	0.02677 (18)	0.0183 (6)	
C2	−0.0206 (2)	0.4780 (2)	0.16074 (18)	0.0165 (6)	
C3	−0.3585 (2)	0.5968 (3)	0.22634 (18)	0.0189 (6)	
C6	−0.1299 (2)	0.5223 (3)	0.28213 (17)	0.0182 (6)	
H6	−0.0996	0.6145	0.3062	0.022*	
C5	−0.2516 (2)	0.5364 (3)	0.19469 (17)	0.0164 (6)	
H5	−0.2787	0.4454	0.1664	0.020*	
C4	−0.2143 (2)	0.6238 (3)	0.12085 (18)	0.0163 (6)	
C7	−0.5546 (3)	0.5477 (3)	0.2590 (2)	0.0270 (7)	
H7A	−0.6337	0.4981	0.2234	0.032*	
H7B	−0.5711	0.6451	0.2465	0.032*	
C8	−0.5230 (3)	0.5208 (3)	0.3643 (2)	0.0334 (7)	
H8A	−0.5017	0.4254	0.3770	0.050*	
H8B	−0.5975	0.5433	0.3851	0.050*	
H8C	−0.4493	0.5761	0.3999	0.050*	
C9	−0.1537 (2)	0.4440 (3)	0.36335 (17)	0.0163 (6)	
C10	−0.1553 (2)	0.5097 (3)	0.44637 (17)	0.0182 (6)	
H10	−0.1368	0.6033	0.4530	0.022*	
C11	−0.1832 (2)	0.4416 (3)	0.51975 (18)	0.0210 (6)	
H11	−0.1870	0.4889	0.5752	0.025*	
C12	−0.2054 (2)	0.3050 (3)	0.51222 (19)	0.0235 (6)	
H12	−0.2225	0.2579	0.5634	0.028*	
C13	−0.2031 (2)	0.2362 (3)	0.43111 (17)	0.0214 (6)	
H13	−0.2176	0.1418	0.4259	0.026*	
C14	−0.1794 (2)	0.3073 (3)	0.35795 (18)	0.0199 (6)	

### Atomic displacement parameters (Å^2^)

**Table:**

	*U*^11^	*U*^22^	*U*^33^	*U*^12^	*U*^13^	*U*^23^
S1	0.0188 (4)	0.0194 (4)	0.0211 (4)	0.0030 (3)	0.0096 (3)	0.0022 (3)
O1	0.0242 (10)	0.0196 (11)	0.0303 (10)	0.0003 (8)	0.0104 (8)	−0.0030 (8)
O2	0.0194 (10)	0.0217 (10)	0.0250 (10)	−0.0018 (8)	0.0098 (8)	0.0004 (8)
O3	0.0205 (10)	0.0177 (10)	0.0216 (10)	−0.0009 (8)	0.0089 (8)	−0.0010 (8)
N1	0.0208 (11)	0.0191 (12)	0.0152 (11)	0.0020 (9)	0.0086 (9)	0.0033 (9)
N2	0.0176 (11)	0.0212 (12)	0.0139 (11)	0.0037 (9)	0.0040 (9)	0.0029 (9)
F1	0.0250 (8)	0.0219 (9)	0.0227 (8)	−0.0002 (7)	0.0035 (7)	−0.0039 (7)
F2	0.0231 (8)	0.0304 (9)	0.0186 (8)	0.0043 (7)	0.0074 (6)	0.0092 (7)
F3	0.0169 (7)	0.0278 (9)	0.0232 (8)	0.0058 (6)	0.0071 (6)	0.0019 (7)
F4	0.0400 (9)	0.0207 (9)	0.0240 (8)	−0.0038 (7)	0.0154 (7)	−0.0038 (7)
C1	0.0189 (13)	0.0156 (13)	0.0225 (14)	0.0026 (11)	0.0097 (11)	0.0005 (11)
C2	0.0163 (13)	0.0144 (13)	0.0189 (13)	−0.0023 (10)	0.0055 (11)	0.0011 (10)
C3	0.0158 (13)	0.0224 (16)	0.0166 (13)	−0.0023 (11)	0.0023 (10)	0.0026 (11)
C6	0.0175 (13)	0.0159 (14)	0.0207 (13)	−0.0004 (11)	0.0052 (11)	0.0001 (11)
C5	0.0185 (13)	0.0155 (13)	0.0155 (12)	0.0016 (11)	0.0058 (11)	0.0004 (10)
C4	0.0170 (13)	0.0136 (13)	0.0189 (13)	0.0016 (10)	0.0066 (11)	−0.0009 (10)
C7	0.0168 (14)	0.0304 (16)	0.0372 (17)	0.0008 (12)	0.0134 (13)	−0.0010 (13)
C8	0.0311 (16)	0.046 (2)	0.0301 (16)	−0.0089 (15)	0.0197 (14)	−0.0049 (14)
C9	0.0127 (12)	0.0202 (14)	0.0156 (12)	0.0032 (11)	0.0041 (10)	0.0031 (11)
C10	0.0144 (13)	0.0177 (14)	0.0207 (14)	0.0010 (10)	0.0030 (11)	−0.0020 (11)
C11	0.0206 (14)	0.0273 (15)	0.0160 (13)	0.0013 (12)	0.0070 (11)	−0.0014 (11)
C12	0.0243 (14)	0.0253 (16)	0.0235 (14)	0.0033 (12)	0.0111 (11)	0.0080 (12)
C13	0.0281 (14)	0.0154 (14)	0.0228 (14)	0.0002 (12)	0.0110 (11)	0.0038 (11)
C14	0.0229 (14)	0.0183 (14)	0.0186 (13)	0.0014 (11)	0.0068 (11)	−0.0032 (11)

### Geometric parameters (Å, º)

**Table:**

S1—C2	1.688 (3)	C6—H6	1.0000
O1—C3	1.207 (3)	C5—C4	1.537 (3)
O2—C3	1.327 (3)	C5—H5	1.0000
O2—C7	1.473 (3)	C7—C8	1.494 (4)
O3—C4	1.411 (3)	C7—H7A	0.9900
O3—H3	0.8400	C7—H7B	0.9900
N1—C2	1.361 (3)	C8—H8A	0.9800
N1—C4	1.442 (3)	C8—H8B	0.9800
N1—H1	0.8800	C8—H8C	0.9800
N2—C2	1.329 (3)	C9—C10	1.384 (3)
N2—C6	1.466 (3)	C9—C14	1.384 (4)
N2—H2	0.8800	C10—C11	1.381 (4)
F1—C1	1.343 (3)	C10—H10	0.9500
F2—C1	1.337 (3)	C11—C12	1.377 (4)
F3—C1	1.335 (3)	C11—H11	0.9500
F4—C14	1.365 (3)	C12—C13	1.377 (4)
C1—C4	1.531 (3)	C12—H12	0.9500
C3—C5	1.510 (3)	C13—C14	1.373 (4)
C6—C9	1.509 (3)	C13—H13	0.9500
C6—C5	1.537 (3)		
			
C3—O2—C7	117.0 (2)	N1—C4—C1	107.66 (19)
C4—O3—H3	109.5	O3—C4—C5	113.1 (2)
C2—N1—C4	124.5 (2)	N1—C4—C5	108.7 (2)
C2—N1—H1	117.8	C1—C4—C5	110.1 (2)
C4—N1—H1	117.8	O2—C7—C8	110.5 (2)
C2—N2—C6	124.1 (2)	O2—C7—H7A	109.6
C2—N2—H2	117.9	C8—C7—H7A	109.6
C6—N2—H2	117.9	O2—C7—H7B	109.6
F3—C1—F2	107.4 (2)	C8—C7—H7B	109.6
F3—C1—F1	106.8 (2)	H7A—C7—H7B	108.1
F2—C1—F1	107.3 (2)	C7—C8—H8A	109.5
F3—C1—C4	111.9 (2)	C7—C8—H8B	109.5
F2—C1—C4	111.5 (2)	H8A—C8—H8B	109.5
F1—C1—C4	111.7 (2)	C7—C8—H8C	109.5
N2—C2—N1	117.7 (2)	H8A—C8—H8C	109.5
N2—C2—S1	120.65 (19)	H8B—C8—H8C	109.5
N1—C2—S1	121.70 (19)	C10—C9—C14	117.0 (2)
O1—C3—O2	125.8 (2)	C10—C9—C6	120.0 (2)
O1—C3—C5	123.3 (2)	C14—C9—C6	122.9 (2)
O2—C3—C5	110.9 (2)	C11—C10—C9	121.2 (3)
N2—C6—C9	112.0 (2)	C11—C10—H10	119.4
N2—C6—C5	107.03 (19)	C9—C10—H10	119.4
C9—C6—C5	112.6 (2)	C12—C11—C10	119.8 (2)
N2—C6—H6	108.4	C12—C11—H11	120.1
C9—C6—H6	108.4	C10—C11—H11	120.1
C5—C6—H6	108.4	C13—C12—C11	120.5 (2)
C3—C5—C6	109.46 (19)	C13—C12—H12	119.7
C3—C5—C4	113.1 (2)	C11—C12—H12	119.7
C6—C5—C4	106.6 (2)	C14—C13—C12	118.4 (3)
C3—C5—H5	109.2	C14—C13—H13	120.8
C6—C5—H5	109.2	C12—C13—H13	120.8
C4—C5—H5	109.2	F4—C14—C13	118.4 (2)
O3—C4—N1	109.9 (2)	F4—C14—C9	118.5 (2)
O3—C4—C1	107.2 (2)	C13—C14—C9	123.0 (2)
			
C6—N2—C2—N1	−0.1 (4)	F3—C1—C4—C5	62.6 (3)
C6—N2—C2—S1	−179.87 (18)	F2—C1—C4—C5	−177.09 (19)
C4—N1—C2—N2	−4.3 (4)	F1—C1—C4—C5	−57.1 (3)
C4—N1—C2—S1	175.49 (18)	C3—C5—C4—O3	54.3 (3)
C7—O2—C3—O1	−1.5 (4)	C6—C5—C4—O3	−66.0 (2)
C7—O2—C3—C5	176.6 (2)	C3—C5—C4—N1	176.62 (19)
C2—N2—C6—C9	157.1 (2)	C6—C5—C4—N1	56.3 (2)
C2—N2—C6—C5	33.2 (3)	C3—C5—C4—C1	−65.6 (3)
O1—C3—C5—C6	69.9 (3)	C6—C5—C4—C1	174.1 (2)
O2—C3—C5—C6	−108.2 (2)	C3—O2—C7—C8	−90.8 (3)
O1—C3—C5—C4	−48.7 (3)	N2—C6—C9—C10	131.1 (2)
O2—C3—C5—C4	133.1 (2)	C5—C6—C9—C10	−108.2 (3)
N2—C6—C5—C3	178.0 (2)	N2—C6—C9—C14	−51.4 (3)
C9—C6—C5—C3	54.5 (3)	C5—C6—C9—C14	69.3 (3)
N2—C6—C5—C4	−59.4 (2)	C14—C9—C10—C11	−0.9 (3)
C9—C6—C5—C4	177.1 (2)	C6—C9—C10—C11	176.7 (2)
C2—N1—C4—O3	98.7 (3)	C9—C10—C11—C12	2.3 (4)
C2—N1—C4—C1	−144.9 (2)	C10—C11—C12—C13	−1.6 (4)
C2—N1—C4—C5	−25.5 (3)	C11—C12—C13—C14	−0.6 (4)
F3—C1—C4—O3	−60.8 (3)	C12—C13—C14—F4	−178.0 (2)
F2—C1—C4—O3	59.5 (3)	C12—C13—C14—C9	2.2 (4)
F1—C1—C4—O3	179.53 (18)	C10—C9—C14—F4	178.7 (2)
F3—C1—C4—N1	−179.01 (19)	C6—C9—C14—F4	1.2 (4)
F2—C1—C4—N1	−58.7 (3)	C10—C9—C14—C13	−1.4 (4)
F1—C1—C4—N1	61.3 (3)	C6—C9—C14—C13	−178.9 (2)

### Hydrogen-bond geometry (Å, º)

**Table:**

*D*—H···*A*	*D*—H	H···*A*	*D*···*A*	*D*—H···*A*
O3—H3···O1	0.84	2.07	2.787 (3)	143
C10—H10···F2^i^	0.95	2.62	3.285 (6)	128
C13—H13···F1^ii^	0.95	2.56	3.262 (8)	131
N1—H1···S1^iii^	0.88	2.52	3.389 (2)	171
N2—H2···O3^iv^	0.88	2.23	3.079 (3)	162

Symmetry codes: (i) *x*, −*y*+3/2, *z*+1/2; (ii) *x*, −*y*+1/2, *z*+1/2; (iii) −*x*, −*y*+1, −*z*; (iv) −*x*, *y*−1/2, −*z*+1/2.
