# Supplementary figures and images for: Crystal structure of ethyl 6-(2-fluoro­phen­yl)-4-hy­droxy-2-sulfanyl­idene-4-tri­fluoro­meth­yl-1,3-diazinane-5-carboxyl­ate
Source: Acta Crystallogr E Crystallogr Commun. 2015 Apr 2;71(Pt 5):o268–9. doi: 10.1107/S2056989015005836 (PMC4420055; doi:10.1107/S2056989015005836)

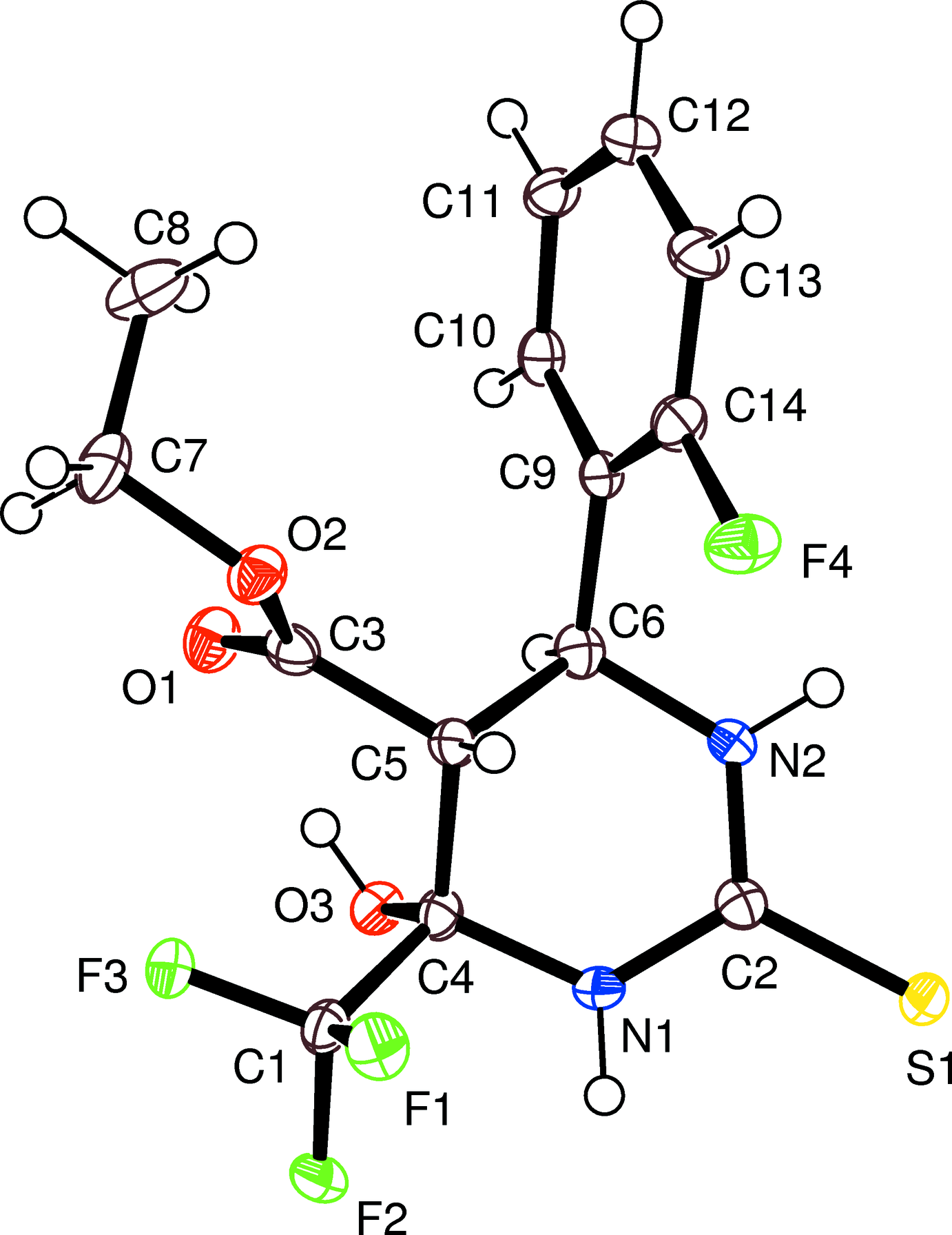

Supplement: Supplementary file 4 [file e-71-0o268-fig1.tif]

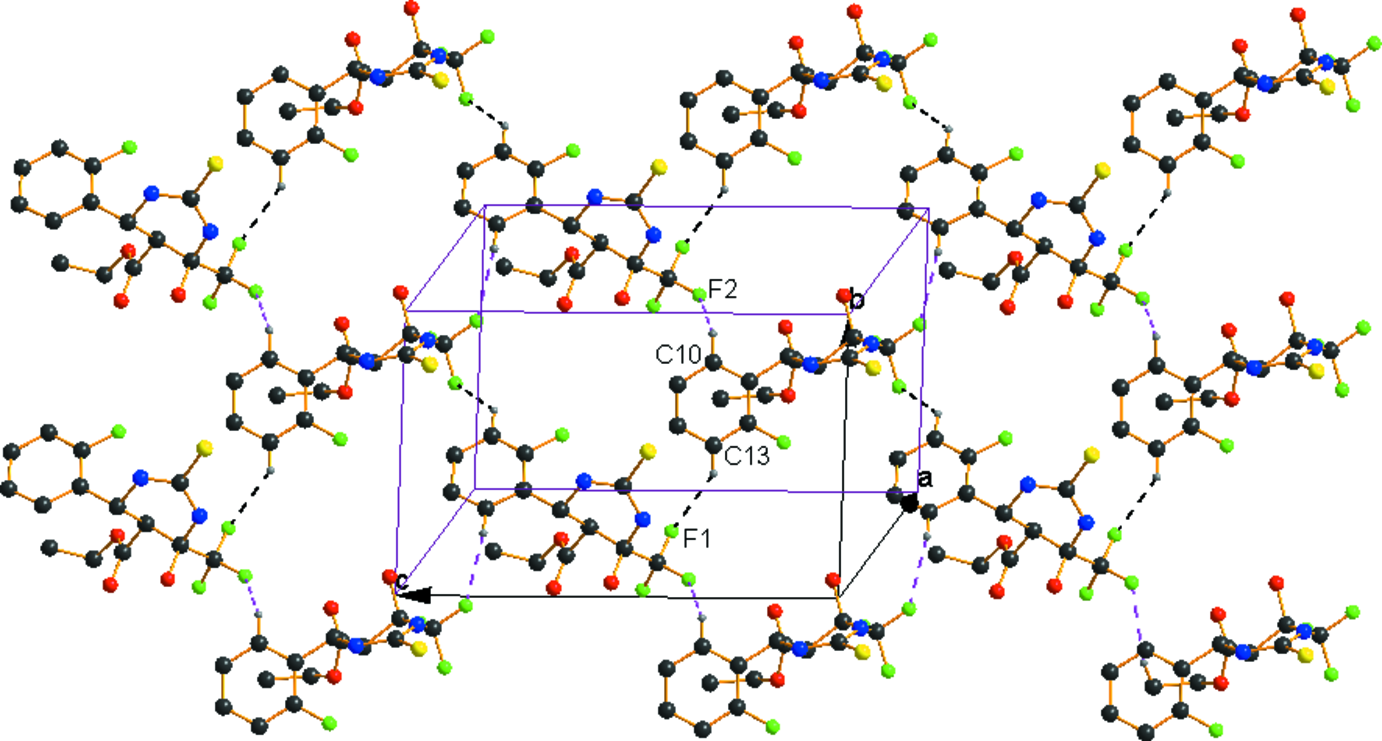

Supplement: Supplementary file 5 [file e-71-0o268-fig2.tif]

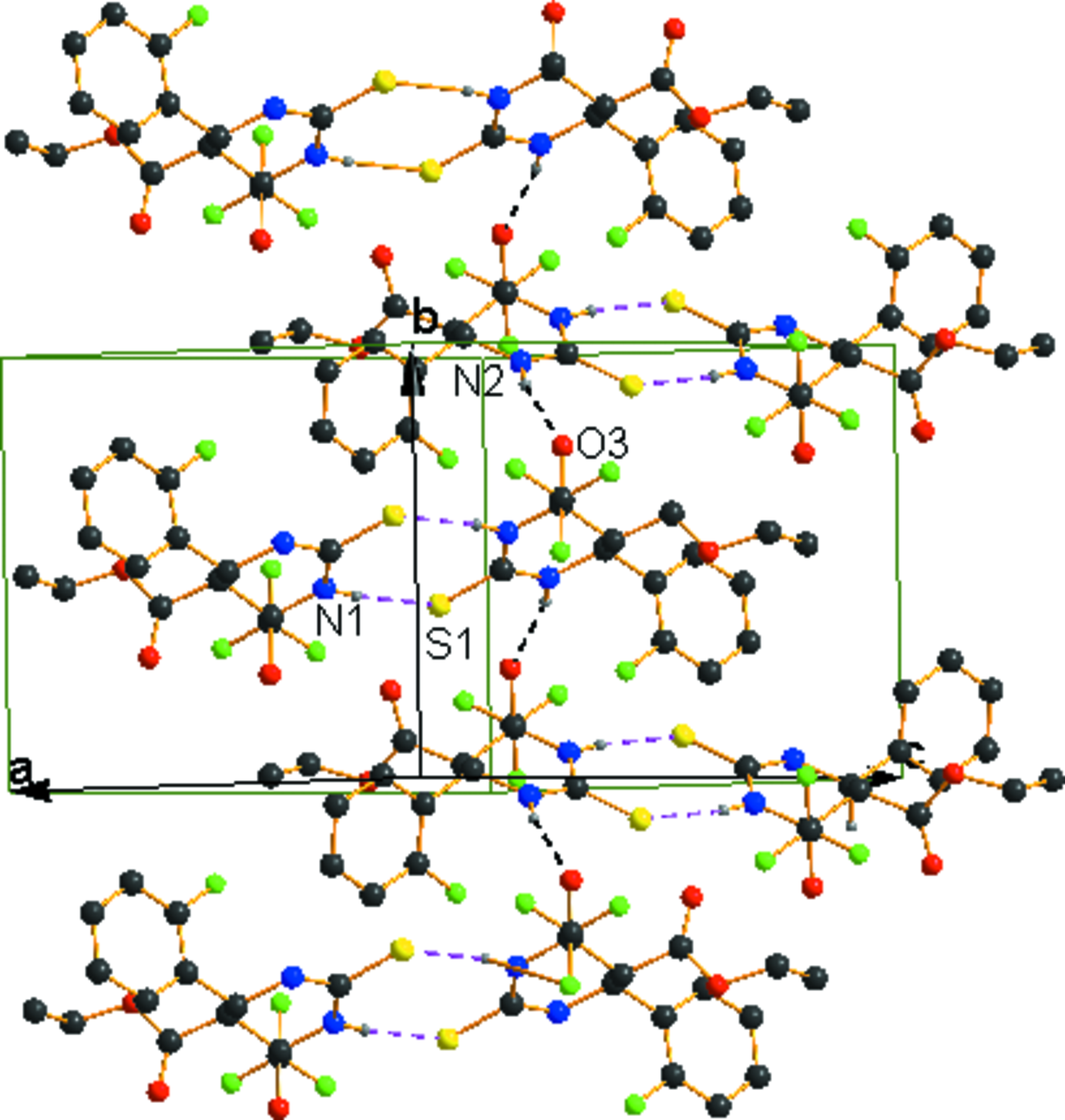

Supplement: Supplementary file 6 [file e-71-0o268-fig3.tif]
